# The Impact of Participants’ Anthropometry on Muscle Activation Levels While Interacting with the Level of Expertise, Task Type, and Single Muscles

**DOI:** 10.3390/jfmk5040088

**Published:** 2020-12-04

**Authors:** Morteza Nagahi, Niamat Ullah Ibne Hossain, Vidanelage L. Dayarathna, Sofia Karam, Kari Babski-Reeves, Raed Jaradat

**Affiliations:** Department of Industrial and Systems Engineering, Bagley College of Engineering, Mississippi State University, Starkville, MS 39762, USA; mn852@msstate.edu (M.N.); ni78@msstate.edu (N.U.I.H.); vld66@msstate.edu (V.L.D.); sk1867@msstate.edu (S.K.)

**Keywords:** construction, carpentry, work-related musculoskeletal disorder, upper extremity, shoulder muscles, electromyography

## Abstract

In this research paper, we implemented a mixed factor design in order to investigate the effect of four anthropometries: height, weight, lower-arm dimensions, and upper-arm dimensions on the muscle activation level of participants when interacting with three types of moderators: experiment expertise, task type, and muscle type. The research paper focused on two levels of expertise (novice and expert), two tasks (deck-building and picket installation), and four arm muscles (Brachioradialis (BR), Extensor Carpi Ulnaris (ECU), Flexor Carpi Radialis (FCR), and Flexor Carpi Ulnaris (FCU)), which resulted in 16 (2 × 2 × 4) groups. For each of the 16 groups, the data were analyzed in order to investigate the relationship between the four anthropometries and the four muscle activation levels of the participants. Amos software (IBM, Armonk, NY, USA), along with multiple group structural equation modeling, was used to test a total of 16 direct relationships, as well as the moderation effects in the designed experiment. The results show that the participants’ expertise can moderate the relationship between their height and muscle activation levels, the relationship between their weight and muscle activation levels, and the relationship between their lower arm dimensions and muscle activation levels. Moreover, the findings of this research paper demonstrate that the relationship between the lower arm dimensions and muscle activation levels, and the relationship between weight and muscle activation levels are moderated by the type of muscle used by the participants (i.e., BR, ECU, FCR, and FCU).

## 1. Introduction

Ergonomics focuses on the different working conditions that affect workers’ physical health and well-being, such as lifting postures, forceful movements, chair and desk designs, room lighting and temperature, and noise [1]. Prolonged work schedules and insufficient breaks between shifts also affect the workers’ comfort. Breaks allow the body to recover from the job, especially if the workers stretch their bodies and exercise, preventing muscles and nerves’ damage, and the development of musculoskeletal disorders (MSDs) [2]. Although ergonomics and human factors both refer to the interlinkage between work conditions and workers’ safety, some say there exists a slight difference between the two [3]. While ergonomics focuses on the impact of the working conditions on workers, human factors investigate the different methods that could help to reduce work injuries and human errors. Due to the increasing number of incidents caused by incorrect postures and excessive heavy lifting, ergonomics has become an indispensable element for laborer’s safety. Addressing the risk factors relevant to the working conditions can help enhance the workers’ safety and prevent injuries [4].

The construction industry encompasses more than 80 distinct jobs [5]. Most of these jobs depend upon manual tasks that are performed by workers (e.g., electricians, construction, carpenters, and others). Construction jobs require great physical efforts, and are fundamental to the US economy. The number of workers in the construction industry was recorded at around 8.82 million in 2017 and increased to 9.07 million in 2018, with a growth rate of 2.74% [6]. Most of the duties performed by the construction workers are heavy lifting and repetitive operations that can cause serious health problems, such as overexertion and MSDs. Work-related injuries (e.g., amputation, broken bones, burns, and paralysis) and health incidents in the construction industry are higher than in other industries (e.g., agriculture, forestry, and fishing). As reported by the US Bureau of Labor Statistics, the construction industry had the most elevated number of injuries, with almost 200,000 injured, more than 1000 seriously injured, and 1008 deaths in 2018 [7]. This is due to the inherent nature of the job itself, which often exposes the workers to hazardous situations where they are at risk of falling, slipping, or tripping if beams and/or roofing collapse; of coming into contact with hazardous substances, equipment and material related injuries; and of transportation-related injuries [8]. Furthermore, incidents related to musculoskeletal disorders (WMSDs) remain the highest among construction workers [9]. WMSDs cause the majority of work-related disabilities, and are responsible for more than 33% of reported injuries in 2016, of which 77% are construction workers [10]. A considerable number of trades related to construction are regarded as high-risk tasks, notably carpentry [11]. Carpenters are required to perform physically-demanding work tasks (e.g., lifting heavy items, insulating buildings, and constructing buildings and bridges), which increases the risk of WMSDs [11]. Studies have shown that 43.5 per 10,000 carpentry workers suffer from exertion [9], which is the highest among similar industries. These injuries are a direct result of the repetitive tasks imposing strain and stress on a certain area of the body, causing acute and sometimes chronic pain.

Previously conducted research reveals the possibility of the existence of a significant difference between experts’ and novices’ muscle activity, and in their corresponding style as they become more skilled [12,13]. Carson and Riek [12] detected changes in muscle activity using Electromyography (EMG) recordings in the upper extremity as the participants became more familiar with their tasks. A study by Babski-Reeves et al. [14] investigated the difference in muscle activity in twelve carpenters (six experts and six novices) while they performed two simulation tasks. The results showed that the level of muscle activity in novices is very high. Another study by Govindu and Babski-Reeves [15] investigated the differences in muscle activity between novices and experts while they performed carpentry tasks. The findings of the study demonstrated that novices worked at high exertion levels for a significant amount of time. These disparities between novices and experts can lead to injuries early in the careers of carpenters. In the same context, research conducted on lifting tasks also concluded that there is a noticeable difference in postures between novice and expert workers, especially for knees and hips [16]. Experts appear to have postures that reduce the force and stress applied to the knees, making them safer from injuries than the novice workers.

One of the important disciplines related to the study of muscle activities, posture improvement, and the prevention of MSDs is anthropometry [17,18]. This is defined as “the science of measurement and the art of application that establishes the physical geometry, mass properties, and strength capabilities of the human body” [19]. Thus, anthropometry is indispensable to the improvement of individuals’ comfort when dealing with designed goods and pieces of equipment [19]. Limb length has implications in the activation of muscles, and this has been studied repeatedly, in particular for gait patterns (e.g., [20,21]). The outcome of these studies indicates that a longer limb lengths result in a larger lever arm, which increases both the internal and external forces and moments at the joint, as well as resulting in increased localized muscle fatigue and activity levels compared to shorter limb lengths. These findings have significant implications, though research comparing limb lengths for the upper extremity is lacking.

While there has been a long-standing interest in anthropometry and its relation to muscle activity, there exists a literary gap concerning the lack of studies to investigate the impacts of anthropometry on muscle activities while considering three moderators, including the level of participants’ expertise, two simulation tasks, and four single muscles.

In order to address this gap, in this research paper, we will study the effect of four anthropometries (height, weight, lower arm, and upper arm) on the muscle activation level of the participants when they interact with three moderators, including the level of expertise, two simulation tasks, and four single muscles. The participants will be asked to undergo a session in which the exposure assessment method surface electromyography (SEMG) is used to collect the data. The objectives of this research paper are summarized as follows:

- To investigate the impact of carpenters’ body dimensions on their muscle activation levels;
- To examine the impact of carpenters’ body dimensions on novice versus expert carpenter’s muscle activation levels;
- To check the impact of carpenters’ body dimensions on the different muscle usages of carpenters;
- To test the impact of carpenters’ body dimensions on their muscle activation levels during two simulated tasks.

Therefore, the following research hypotheses have been defined to be investigated in this research paper.

**Hypothesis** **1.** *Does the participant’s Height affect the four measures of muscle activation level?*

**Hypothesis** **2.** *Does the participant’s Weight influence the four measures of muscle activation level across the 16 designed groups?*

**Hypothesis** **3.** *Does the participant’s Lower arm influence the four measures of muscle activation level?*

**Hypothesis** **4.** *Does the participant’s Upper arm affect the four measures of muscle activation level across the 16 designed groups?*

## 2. Materials and Methods

### 2.1. Data Collection Procedure and Participants

The participants were given a written and verbal description of the research, informed consent documentation approved by the Mississippi State University Institutional Review Board for the Protection of Human Subjects in Research (protocol number of IRB-12-401, approved on December 2012) office prior to any experimentation, and the detailed objectives of the research paper. Demographic questionnaires were completed by the participants, and then their inclusion was determined. All of the participants were given training on how to use the Borg Scale [22]. This training required the participants to squat along the wall with their legs at 90 degrees and then step through the Borg scale until they reached exhaustion. Before the task was started, the participants were given the required data collection equipment, and the novices were trained to understand the task protocols. After 15 min into the task, the participants were seated, and they were asked to provide a Borg rating after 10 min of rest. The participants could only continue to the second task if their Borg ratings were one or less. If their rating was more than one, the participants’ rating was checked every minute until it reached one or lower. After the second task was completed, the participants were compensated for their time.

A total of 42 participants were analyzed as a part of a larger study. Twenty-one participants were experts, while the other half were novices. The number of females and males recruited were representative of the construction industry (novices: 17 males and 4 females; experts: 20 males and 1 female). All of the participants had to meet the criteria of being free from any musculoskeletal injuries to their upper extremity. A demographic questionnaire was circulated in order to obtain this information via self-report. Table 1 shows the demographic characteristics of the sample population.

### 2.2. Experimental Design

A mixed factors design was used to test the effects of the participants’ anthropometry—height, weight, lower arm, and upper arm—on their muscle activation levels while interacting with moderators: participants’ expertise (two levels of novice and expert), types of the task (deck building and picket installation), and types of upper-arm muscle (BR, ECU, FCR, and FCU). Initially, we classified the data into 16 groups based on a designed experiment. The experimental design includes two levels of participants’ expertise (novice and expert), two levels of the task type (deck-building and picket installation), and four different muscles (Brachioradialis (BR), Extensor Carpi Ulnaris (ECU), Flexor Carpi Radialis (FCR), and Flexor Carpi Ulnaris (FCU)). Then, we assessed the impact of the participants’ anthropometry on their muscle activation levels. These two tasks (picket installation and deck building) and the upper-arm muscles were selected based on discussions with a residential construction carpenter. The principal investigator was invited to observe and record the activities required for the performance of several finished carpentry tasks. Following these observations, two tasks (picket installation and deck building) were mocked up for the study. These two activities were selected as the tasks for the mock-up, as these could be completed without assistance from other carpenters and require few tools.

### 2.3. Data Analysis Method

In this research paper, we used multiple-group structural equation modeling using AMOS software v24.0 (IBM, Armonk, NY, USA). There are many advantages with this methodology, such as Structural Equation Modeling (SEM), which would help: (1) to explain the simultaneous relationships between all of the research variables, including the Independent Variables (IVs), Dependent Variables (DVs), and moderators (which are not step by step and independent from each variable); (2) to include multiple DVs at the same time (in this research paper, there are four DVs: the mean of the average, the median of the average, the mean of the peak, and the median of the peak of the SEMG based muscle activation level); (3) to account for any measurement error associated with each DV separately (four error terms associated with the four DVs were included); (4) to build a complex theoretical map that shows the big picture of the interrelationships among all of the research variables (our theoretical model shows 256 simultaneous relationships); (5) to obtain comprehensive model-fit indices that help us to conduct a thorough construct validity; (6) to conduct advanced analytical techniques, such as multiple group structural equation modeling. These features and advantages are necessary in order to obtain valid and reliable results.

### 2.4. The Proposed Theoretical Model

**Figure 1:**
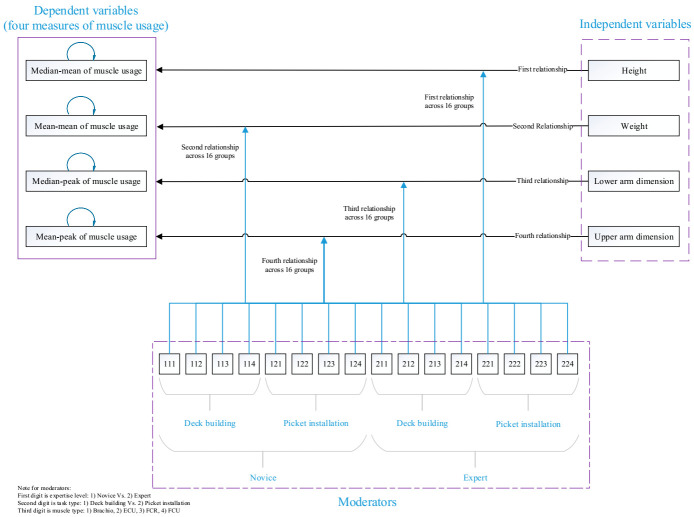
The proposed theoretical model of the research paper.

A mix factor design was selected in order to examine the effect of the four anthropometries—including height, weight, lower-arm dimension, and upper arm—on the muscle activation level of the participants when they interact with three types of moderators: experiment expertise, task type, and muscles. The research paper focused on two levels of expertise (novice and expert), two types of tasks (deck-building and picket installation), and four levels of the arm muscle: BR, ECU, FCR, and FCU, which resulted in 16 (2 × 2 × 4) groups. For each of the 16 groups, the data were analyzed in order to investigate the relationship between four anthropometries and four muscle activation levels of the participants, which resulted in 256 simultaneous relationships among the research variables (see Figure 1). Figure 1 shows the proposed theoretical model of this research paper.

### 2.5. Research Variables and Measures

#### 2.5.1. Independent Variables

The theoretical model consists of four main independent variables which are relevant to the participants’ anthropometry, namely, (1) height, (2) weight, (3) lower arm, and (4) upper arm.

#### 2.5.2. Dependent Variables

Four SEMG-based measures of muscle activation level—(1) the mean of the average, (2) the median of the average, (3) the mean of the peak, and (4) the median of the peak—were designed as the four dependent variables of this research paper.

#### 2.5.3. Moderators

Three moderators were used in this research paper: (1) participants’ expertise, (2) type of simulation task, and (3) four different arm muscles. Detailed information about moderator variables is discussed below.

##### Participant Experience Level

The experience level for each of the participants was categorized as ‘novice’ or ‘expert’. A group of college students (*n* = 21) was asked to participate as the novice participants for this research paper. These students did not have any previous experience in construction or any hobbies related to residential construction. The expert-level participants (*n* = 21) were residential construction carpenters who had years of hands-on experience. These workers had to meet the following criteria:

1. Have three or more years of construction work experience.
2. Have not missed more than three months of work in the previous year.
3. Two of their three years of experience must have been as a carpenter.
4. Have a minimal injury history, and their work time loss must be less than four days missed per year due to work-induced discomfort/injuries.

While it is recognized that students and/or persons with no carpentry experience may not represent a true match with workers who are new to construction (with respect to motivation, etc.), this approach has been used in previous studies (e.g., [23,24]), and has been found to successfully identify measurable and observable differences between expert and novice participants (the objective of this research). Furthermore, as this is an exploratory study into the differences in upper extremity intensive tasks, this was considered acceptable. Any follow-up on this study should include apprentice carpenters. The work time loss must be less than four days missed per year due to work-induced discomfort/injuries.

##### Work Task Simulations

**Figure 2:**
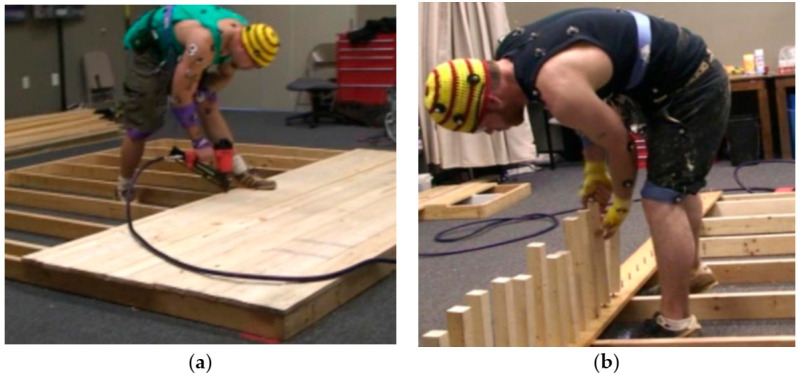
Laboratory task simulations: (**a**) deck building (left) and (**b**) picket installation.

Based on a consultation with a residential construction carpenter, two tasks—namely picket installation and deck building—were chosen for this research paper. The PI was invited to observe and record the activities required for the performance of several finished carpentry tasks. Following these observations, and in consultation with a residential construction carpenter, two tasks (picket installation and deck building) were mocked up for the study. The two activities were selected as tasks for the mock-up because they could be completed without assistance from other carpenters, and require few tools. Each of the participants completed two tasks for a 15-min time frame, with a total of 30 min of work. The first task was deck building, and the second task was picket installation, as shown in Figure 2. The participants received an explanation of both tasks and a 5-min familiarization period (followed by five minutes of rest) to become accustomed to using a nail gun. The participants’ pace of work was self-determined. There was a 15-min break period that was designated to minimize the fatigue associated with task performance. The minimum time of rest allowed between each task was 10 min. Before beginning the second work task, the participants were asked to verbally rate their perceived level of fatigue using a modified Borg CR-10 Perceived Level of Exertion Scale [22]. None of the participants were allowed to proceed to the second task until a perceived fatigue level of one or lower was reported. This was carried out in order to reduce any confounding influence of fatigue on task performance. All of the tests were completed within a single test session lasting approximately two hours.

During the picket installation (first task), a picket was used to screw the present lags along with the perimeter of a deck plank (Figure 2b). The pickets were 1 ¾″ long, with pre-drilled holes at the end of each for ease of attachment. The participants manually screwed the pickets onto the lags until they were tight and flush with the deck plank. Prior to starting the task, a member of the research team pre-attached the deck plank to the deck. All of the necessary materials for this task were made available to the participants.

For the deck building task (second task), the participants were tasked to install deck planks on a 10’ × 10′ surface (Figure 2a). Each participant secured the board with two nails at each end and each joist of the decking. One of the planks—which had a pre-inserted lag—was to be secured first on the outside of the deck frame. All of the required materials for this task were given to the participants.

##### Four Arm Muscles and SEMG Measurement

Four different muscles: Brachioradialis (BR), Extensor Carpi Ulnaris (ECU), Flexor Carpi Radialis (FCR), and Flexor Carpi Ulnaris (FCU), were selected in this study. The surface electromyography (SEMG) of select forearm muscles was used to analyze the differences in the muscle activation strategies during performance of the task. The surface electromyography (SEMG) measurements of the ECU, BR, FCU, and FCR were obtained using a circular, 10-mm Ag/AgCl pre-gelled bipolar disposable electrode. Prior to the electrode’s application, the entire skin surface area was shaved, slightly abraded, and cleansed with alcohol in order to guarantee minimal impedance. The electrodes for the FCU were located 3 cm medial to the ulna at a point one-third of the length of the forearm from the elbow crease [25]. The electrodes used for the ECU were located one-third of the distance between the lateral epicondyle of the humerous and the olecranon process and the styloid process of the ulna [26]. The electrodes for the FCR were located three to four fingerbreadths distal from the midpoint of a line connecting the medial epicondyle and the biceps of the humerous, immediately medial to the midpoint of a line connecting the medial epicondyle and biceps tendon [25]. For the BR, the electrodes were located at the lateral epicondyle of the humerous, immediately medial to the brachioradialis muscle [25]. The inter-electrode distance was then set to 2.5 cm. The signals were then transmitted through short (less than 30 cm) leads to preamplifiers (×100 gain). Next, the leads were secured to the arm with tape in order to reduce noise and decrease displacement. The surface electromyography (SEMG) signals were hardware amplified, band-pass filtered (10–500 Hz), Root Mean Square (RMS) converted (110 ms time constant), and A/D converted. The amplifier gain was set so that the signals could not exceed 2–3 volts. The input impedance was measured using a standard voltmeter in order to ensure impedance was within acceptable levels (<10 kΩ).

After the stabilization of the electrodes (15 min), resting and maximum voluntary contractions (MVCs) were obtained. The resting RMS SEMG measurements were recorded at 256 Hz for six seconds with the participant’s hands in their lap or resting along their sides. The MVCs were performed in representative postures (for each simulated task) in order to improve the accuracy of the MVC readings. For the deck-building task, the MVCs held a dynamometer [27] with the upper arm resting along the side of the body, with the elbow bent at 90 degrees and the wrist straight. We recognize that a study by Juul-Kristensen et al. [28] showed that traditional handgrip strength tests using dynamometers underestimated MVC by an average of 34% for the participants tested. However, the posture assumed while holding the dynamometer closely represents the posture used to hold a hammer. For the stair picket exertion task, the participants grasped a ¾” cylinder and twisted with both hands in opposite directions. This simulated the action of screwing stair pickets into the floor surface of the staircase. The fixtures and postures were constructed in order to isolate the muscles being investigated. Three MVCs were generated for each muscle independently, each lasting about three seconds, with a one-minute rest period between each. In the event that the third MVC resulted in a maximum, additional MVCs were performed until the subsequent MVCs were less than the maximum. A number of studies have used handgrip strength tests to obtain MVC data for the forearm muscles, and these were also used in this research paper. The participants’ Peak RMS SEMG signals were identified for each trial using Noraxon’s MyoResearch XP Master Edition software (Scottsdale, AZ, USA), and the maximum value was taken as the MVC for that muscle for the normalization of the task SEMG.

The task RMS SEMG measurements were sampled at 256 Hz for the entire test session using a Noraxon’s Myosystem 1400A (Scottsdale, AZ, USA), and subsequently smoothed (using a 5 Hz low pass filter) and stored. This smoothed data were used to estimate the normalized force levels. The peak and mean RMS values were calculated for the entire test session, and at 30-s increments (for the trend analysis). The first and last 10 s of the experimental condition were removed in order to reduce the start-up and task completion effects. The processed data were expressed in terms of percent MVC.

## 3. Results

We investigated the simultaneous impact of four main independent variables (anthropometry)—namely, (1) height, (2) weight, (3) lower arm, and (4) upper arm—on four dependent variables, including (1) the mean of the average, (2) the median of the average, (3) the mean of the peak, and (4) the median of the peak of the SEMG-based muscle activation level. Then, we compared these 16 (4 IVs × 4 DVs) relationships for each of the 16 identified groups (e.g., the BR muscle activation level of a novice participant working on a picket installation task, or—as another example—the FCU muscle activation level of an expert participant working on deck building task). As a result, we tested 256 (16 × 16) relationships in the designed experiment. Summaries of the significant relationships are presented in Table 2, Table 3, Table 4 and Table 5. Each table shows the impact of one particular independent variable on four dependent variables (four muscle activation level measures) across 16 different groups. For example, one group could be the BR muscle activation level of a novice participant working on the picket installation task.

### 3.1. Findings of the First Hypothesis

The first hypothesis was tested along with its sub-hypotheses, and the results showed the impact of the participants’ height on four muscle activation level measures across 16 groups (see Table 2). Based on the corresponding results, the participants’ height has a significant inverse impact on individuals’ muscle activation level in 10 out of 16 groups. In other words, shorter people use more muscle power than taller participants do. In the presence of moderators, interesting findings were observed, as listed below:

1. The relationship between height and muscle activation level is moderated by participants’ expertise. Height only influences the FCR muscle activation level in the expert sample, while all four muscle activation levels of the novice participants are influenced by height. The reverse impact of height on muscle activity is stronger for novices than experts.
2. The relationship between height and muscle activation level is not moderated by the simulation task. The impact of height on the muscle activation level in the deck-building task is not significantly different from the picket installation task. On the other hand, BR and ECU usage are better predictors of the deck building task than the picket installation task, while FCR and FCU are better predictors of the picket installation task than the deck building task.
3. The relationship between height and muscle activation level is not moderated by the type of the single muscle. However, the impact of height on the muscle activation level is not significantly different across single muscles, and this relationship is stronger for FCR and BR usage.

### 3.2. Findings of the Second Hypothesis

Table 3 shows the second main hypothesis of the research paper and indicates the influence of the participants’ weight on the four muscle activation level variables across 16 groups. Based on the corresponding result, the participants’ weights have a significant positive correlation with the muscle activation level in eight out of 16 groups. In other words, heavier participants use more muscle power than lighter participants do. In two groups out of 16, the relationship is negative. In the presence of the moderators, the following interesting findings were observed:

1. The relationship between weight and muscle activation level is moderated by participants’ expertise. The overall relationship between weight and muscle activation level is different for experts and novices, especially with regard to the ECU muscle.
2. The relationship between weight and muscle activation level is not moderated by the simulation task. The impact of weight on muscle activation level in the deck-building task is not significantly different from the picket installation task; however, this relationship is stronger for the picket installation than the deck building task.
3. The relationship between weight and muscle activation level is moderated by the type of the single muscle. The impact of weight on the muscle activation level is significantly different across single muscles, and this moderation effect is stronger for the FCR and ECU muscles, and is the weakest for the FCU muscle.

### 3.3. Findings of the Third Hypothesis

Table 4 shows the result of the testing of the third hypothesis and exhibits the effect of the participants’ lower arm dimensions on four muscle activation level measures across 16 groups. Based on the corresponding results, the participants’ lower arm dimensions have a reverse impact on the muscle activation level in 12 out of 16 groups. In other words, people with small lower arm dimensions use more muscle power than other participants. In the presence of the moderators, the following interesting findings were observed:

1. The relationship between the lower arm dimensions and the muscle activation level is moderated by the participants’ expertise. The overall relationship between the lower arm and the muscle activation level is stronger for novices than experts.
2. The relationship between the lower arm dimensions and the muscle activation level is not moderated by the simulation task. The impact of the lower arm on the muscle activation level in the picket installation task is stronger than in the deck-building task, especially for the FCU and BR muscles.
3. The relationship between the lower arm dimensions and the muscle activation level is moderated by the type of the single muscle. The impact of lower-arm dimensions on the muscle activation level is significantly different among single muscles, especially among experts.

### 3.4. Findings of the Fourth Hypothesis

Table 5 displays the relationship between the participants’ upper arm dimensions and four muscle activation level measures across 16 groups. Based on the corresponding results, the participants’ upper arm dimensions do not have a significant impact on the muscle activation levels in almost all of the groups. In other words, the participants with small upper-arm dimensions are not different from their counterparts in regard to muscle activity. Since the upper arm does not affect the muscle activation level, the further investigation of this relationship in the presence of moderators was not carried out.

## 4. Discussion

### 4.1. Consistency with the Literature

The literature shows that novice and expert workers are significantly different when completing work tasks. For instance, there were significant differences found in material handling tasks [16,29,30,31] and in sports activities [32,33,34]. The usage of different single muscles during work tasks can be varied depending upon the workers’ task type and expertise. An experimental study performed by Govindu et al. [35] showed that experts use the FCU and FCR muscles for a higher percentage of the task times, while novices use ECU muscles for the majority of the task times. Several other studies have also shown that the usage of different single muscles between novice and expert workers is significantly different, particularly in the carpentry and construction field [15,36,37]. Another stream of research used deck building and picket installation tasks to compare the ways in which the muscle activation level and expertise level matter in a working task [14,15,35]. For instance, a study conducted by Babski-Reeves et al. [14] found that the forearm muscular loading of novice carpenters was not significantly different between deck building and picket installation.

### 4.2. Hypothesis 1 Interpretation

The existing literature provides strong evidence that height has an impact on the muscle strength and usage of people during a task [38,39,40]. In order to investigate the impact of height on handgrip strength, Hogrel et al. [38] conducted a study with 96 healthy children. The outcome of the study indicated that height is a strong predictor for handgrip strength in children. Niempoog et al. [39] and Jürimäe et al. [40] also confirmed that hand muscle strength is highly correlated with body height during a work task. The finding of our research paper is consistent with the previous literature; however, it exhibits a negative correlation between height and four arm muscles (BR, ECU, FCR, and FCU). This result implies that the shorter participants of the research use excessive muscle power compared to taller people to get the job done. The results highlight the important fact that shorter workers may suffer more work-related musculoskeletal disorders (WMSDs). Moreover, the height of novice participants has a higher negative correlation with muscle activity compared to the experts in the research sample. Based on the results, H_1a_ (participants’ expertise moderate the impact of the Height on muscle usage) is supported. However, H_1b_ (task type moderate the impact of the Height on muscle usage) and H_1c_ (muscle type moderate the impact of the Height on muscle usage) are not supported, as the task type (Deck and Picket) or type of single muscle do not moderate the impact of height on the muscle activation level.

### 4.3. Hypothesis 2 Interpretation

Body size was found to be a major factor that defines individuals’ muscle strength, while lower weight is associated with poor muscle strength, and vice versa [41,42]. A comprehensive study conducted by Era et al. [41] concluded that body mass positively correlated with the isometric strength of knee extension, elbow flexion, trunk extension, and handgrip of 75+ years old female adults. In another study that measured handgrip strength, Rantanen et al. [42] found that higher body weight is associated with hypertension, as muscle strength is lower in lightweight people. The results of our research paper confirmed that participants’ weight has a positive impact on muscle strength, as is consistent with previous literature. Moreover, an experimental study conducted by Davis et al. [43] showed that “heavier athletes would require more force to generate movement,” which confirmed our finding. The novice and expert participants showed a significant difference with respect to the relationship between weight and muscle activation level in this research paper. Additionally, the type of the single muscle moderates the association between weight and muscle activation level. Based on the results, H_2a_ (participants’ expertise moderate the impact of the Weight on muscle usage) and H_2c_ (muscle type moderate the impact of the Weight on muscle usage) are supported. H_2b_ (task type moderate the impact of the Weight on muscle usage) is not supported, as the task type does not moderate the impact of weight on muscle activation level.

### 4.4. Hypothesis 3 Interpretation

The finding of the research paper shows that the participants’ lower arm length is negatively correlated with their muscle activation level. This means that muscle power usage is higher for people with a smaller lower arm than people with larger lower-arm dimensions. These findings are contrary to what was found for lower limb differences and gait studies. This discrepancy is likely due to the fact that the participants can accommodate limb differences by adjusting their posture. There were also large differences between the limb lengths reported for the novices and experts. There is no explanation for this large difference, but these differences would have potentially large impacts on any differences between the expert and novice participants. The expert participants showed a stronger relationship between the lower arm and muscle activation level than novices. The type of single muscle was found to moderate the association between the lower arm and muscle activation level. The relationship is greater in the expert group than for the novices. Consistent with our results, Govindu et al. [35] detected that experts heavily use the FCU and the FCR muscles, while novices use the ECU muscle at a greater percentage during a task. Based on the results, H_3a_ (participants’ expertise moderate the impact of the Lower-arm dimension on muscle usage) and H_3c_ (muscle type moderate the impact of the Lower-arm dimension on muscle usage) are supported. H_3b_ (task type moderate the impact of the Lower-arm dimension on muscle usage) is not supported, since the simulation task does not have a moderation effect on the relationship between the lower arm and muscle activation level.

### 4.5. Hypothesis 4 Interpretation

The results of the research paper indicate that there is no significant relationship between participants’ upper arm dimensions and muscle activation level. This means that the size of the upper arm does not affect muscle activity during a work task. Since the impact of the upper arm on the muscle activation level is not significantly different, H_4a_ (participants’ expertise moderate the impact of the Upper-arm dimension on muscle usage), H_4b_ (task type moderate the impact of the Upper-arm dimension on muscle usage), and H_4c_ (muscle type moderate the impact of the Upper-arm dimension on muscle usage) are not supported.

The kinematic assessment focused on the entire upper extremity, even though the SEMG only quantified the forearm muscle activity. Ideally, more muscles would have been measured. However, the forearm was chosen for two reasons. First, it was chosen due to the loading that was placed on the musculature in order to perform the necessary activities. The second reason it was chosen was that future studies could investigate a complete musculature. The muscles selected also were coordinated pairs of muscles (extensor and flexor muscles that work in coordination with each other for various hand/arm movements). Therefore, as one set of muscles is contracting, the other set needs to relax in order to support that movement. This would impact the activation patterns of each pair of muscles, and over time would impact the fatigue that the individual muscle would experience. This study did not collect data for a sufficiently long period of time to understand the fatigue characteristics of these tasks, but the higher levels of activation by the novices would result in greater muscle fatigue rates. Furthermore, as muscle force is directly related to intramuscular coordination (the ability of the individual muscle fibers within a muscle to fire synchronously), higher muscle activity levels may be indicative of higher levels of intramuscular coordination.

## 5. Conclusions

The purpose of this research paper was to identify the differences in the muscle activation level measurements between novices and experts performing simulations of residential carpentry tasks while considering four (BR, ECU, FCR, and FCU) muscles. These relationships differed across novice versus experts, deck building versus picket installation, and the four single muscles. For instance, height has a stronger effect on the muscle activation level for novice participants than experts, and the relationship between the lower arm dimensions and the muscle activation level is weaker for experts. The type of task performed does not affect the relationship between height and muscle activation level, while the relationship is stronger for picket installation when it comes to the weight measure. Comparing the overall results of the four tables (Table 2, Table 3, Table 4 and Table 5), we can observe that participants’ lower arm (Table 4) is the most important factor in predicting the participants’ muscle activation level in this research paper. In other words, the participants’ lower arm has a significant impact on participants’ muscle activation levels in the different experimental designs. Additionally, the participants’ weight and height are important factors that affect muscle activities. On the other hand, participants’ upper arms (Table 5) have little to no impact on the participants’ muscle activation levels in different experimental designs.

The simulation of the work tasks in the laboratory eliminates exposures to environmental and real-world conditions that may affect work task parameters (such as order, etc.). This is an inherent limitation of all laboratory studies. While this is recognized, it is not considered to sufficiently detract from the potential gains in knowledge that can be obtained from the successful completion of this project. A further limitation of the simulations is the limitation of the time, as the participants had to perform the work tasks within a shorter period of time for this research paper. A 15-min time period was selected to collect the preliminary data on the differences in the novice and expert work strategies, while at the same time minimizing the effects of fatigue on the workers’ strategies. Furthermore, this time period will allow for a preliminary investigation into adaptation and learning for novice workers over a brief interval. Future studies should investigate the differences for longer periods of time.

Injuries and illnesses were excluded in this research paper, as the focus was on the upper extremity. The selection of the upper extremity was to assist in scoping the research paper to a manageable level. It is understood that other body parts might significantly be affected by WMSDs, and should be studied, but they are considered outside the scope of this research paper.

Additionally, all of the trades within construction were not studied and evaluated. Carpentry was chosen based on reviews of epidemiology in construction. Again, the selection of a single trade (that is, carpentry) was the scope of the research paper, and is in line with the R03 guidelines. Future studies could be conducted in order to investigate a number of trades within construction. The pilot and exploratory nature of the research underlies the experimental design. If the proposed work is effective for the selected trades, it is expected that it would be effective for other trades with similar types of work tasks. Further investigations on the trainability of professional practices, as have been carried out for MMH/lifting tasks, could be developed for picket installation and deck building tasks based on the results of this research paper.

## Figures and Tables

**Table 1:** Demographic characteristics of the sample population.

Variables		Novice M (*SD*)	Expert M (*SD*)
Age (years)		24.3 (5.9)	34.5 (11.5)
Carpentry experience (years)		0.003 (0.017)	13.1 (9.6)
Construction experience (years)		0.125 (0.3)	12.8 (8.9)
Weight (Kg)		82.2 (22.6)	82.4 (12.7)
Height (M)		1.770 (0.08)	1.80 (0.09)
Upper Arm (M)		0.337 (0.025)	0.770 (0.094)
Lower Arm (M)		0.199 (0.014)	0.193 (0.020)
Gender	Male	17	20
	Female	4	1

**Table 2:** Impact of Height on the measures of muscle activation levels (that is, the mean of the average, the median of the average, the mean of the peak, and the median of the peak) across 16 groups.

Groups			Height Has a Significant Impact on the Following Measure of Muscle Activation Level	Unstandardized Coefficient	Standard Error	*t*-Value	Standardized Coefficient
Expertise	Task	Muscle					
Novice	Deck	BR	Median of average	−0.029 ***	0.010	−2.938	−0.740 ***
			Mean of average	−0.026 ***	0.009	−2.776	−0.708 ***
			Median of the peak	−0.102 ***	0.039	−2.640	−0.689 ***
			Mean of the peak	−0.108 ***	0.040	−2.732	−0.704 ***
		ECU	Median of average	−0.030 ****	0.007	−4.332	−0.908 ****
			Mean of average	−0.029 ****	0.006	−4.559	−0.934 ****
			Median of the peak	−0.082 ***	0.031	−2.701	−0.672 ***
			Mean of the peak	−0.085 ***	0.034	−2.507	−0.632 ***
		FCR	Median of average	−0.074 ****	0.019	−3.992	−0.894 ****
			Mean of average	−0.075 ****	0.020	−3.833	−0.882 ****
			Median of the peak	−0.349 ****	0.095	−3.684	−0.878 ****
			Mean of the peak	−0.390 ****	0.100	−3.879	−0.909 ****
		FCU	Median of average	−0.034 **	0.016	−2.079	−0.586 **
			Mean of average	−0.034 **	0.017	−2.046	−0.581 **
			Median of the peak	−0.123 *	0.064	−1.904	−0.551 *
			Mean of the peak	−0.119 *	0.063	−1.887	−0.543 *
	Picket	BR	Median of average	−0.020 ***	0.007	−2.986	−0.710 ***
			Mean of average	−0.021 ***	0.007	−3.070	−0.726 ***
			Median of the peak	−0.103 ***	0.035	−2.976	−0.666 ***
			Mean of the peak	−0.098 ***	0.033	−2.959	−0.662 ***
		ECU	Median of average	−0.035 ****	0.009	−3.822	−0.831 ****
			Mean of average	−0.035 ****	0.009	−3.870	−0.829 ****
			Median of the peak	−0.130 ****	0.054	−2.387	−0.593 ****
			Mean of the peak	−0.134 ****	0.055	−2.457	−0.600 ****
		FCR	Median of average	−0.073 ****	0.014	−5.423	−0.953 ****
			Mean of average	−0.075 ****	0.014	−5.186	−0.939 ****
			Median of the peak	−0.435 ****	0.087	−4.994	−0.935 ****
			Mean of the peak	−0.510 ****	0.105	−4.851	−0.916 ****
		FCU	Median of average	−0.018 **	0.008	−2.405	−0.653 **
			Mean of average	−0.018 **	0.008	−2.323	−0.636 **
			Median of the peak	−0.093 **	0.038	−2.423	−0.644 **
			Mean of the peak	−0.094 **	0.038	−2.491	−0.656 **
Expert	Deck	BR	-	-	-	-	-
		ECU	-	-	-	-	-
		FCR	Median of average	−0.015 **	0.007	−2.187	−0.552 **
			Mean of average	−0.016 **	0.007	−2.108	−0.546 **
			Median of the peak	−0.163 **	0.067	−2.427	−0.643 **
			Mean of the peak	−0.185 **	0.071	−2.588	−0.667 **
		FCU	Median of average	0.007 *	0.004	1.700	0.089 *
	Picket	BR	-	-	-	-	-
		ECU	-	-	-	-	-
		FCR	Median of average	−0.020 **	0.008	−2.561	−0.648 **
			Mean of average	−0.020 **	0.008	−2.557	−0.644 **
			Median of the peak	−0.190 **	0.077	−2.450	−0.643 **
			Mean of the peak	−0.190 **	0.073	−2.591	−0.666 **
		FCU	-	-	-	-	-

*: *p* < 0.1; **: *p* < 0.05; ***: *p* < 0.01; ****: *p* < 0.001.

**Table 3:** Impact of Weight on four muscle activation level measures across 16 groups.

Groups			Weight Has a Significant Impact on Muscle Activation Level	Unstandardized Coefficient	Standard Error	*t*-Value	Standardized Coefficient
Expertise	Task	Muscle					
Novice	Deck	BR	Median of average	0.005 *	0.003	1.708	0.088 *
		ECU	Median of average	0.001 ***	0.001	2.787	0.651 ***
			Mean of average	0.002 ***	0.001	3.082	0.703 ***
			Median of the peak	0.004 *	0.002	1.793	0.497 *
			Mean of the peak	0.004 *	0.003	1.665	0.467 *
		FCR	Median of average	0.003 **	0.001	2.417	0.603 **
			Mean of average	0.004 **	0.002	2.322	0.595 **
			Median of the peak	0.017 **	0.007	2.298	0.610 **
			Mean of the peak	0.020 **	0.008	2.565	0.670 **
		FCU	-	-	-	-	-
	Picket	BR	Median of average	0.001 **	0.001	1.982	0.525 **
			Mean of average	0.001 **	0.001	2.055	0.541 **
			Median of the peak	0.005 *	0.003	1.798	0.448 *
			Mean of the peak	0.005 *	0.003	1.889	0.471 *
		ECU	Median of average	0.002 **	0.001	2.118	0.513 **
			Mean of average	0.001 **	0.001	2.117	0.505 **
		FCR	Median of average	0.004 ****	0.001	3.909	0.846 ****
			Mean of average	0.004 ****	0.001	3.767	0.841 ****
			Median of the peak	0.023 ****	0.007	3.443	0.795 ****
			Mean of the peak	0.028 ****	0.008	3.394	0.792 ****
		FCU	-	-	-	-	-
Expert	Deck	BR	-	-	-	-	-
		ECU	Median of average	−0.001 **	0.001	−2.056	−0.439 **
			Mean of average	−0.001 **	0.001	−2.011	−0.434 **
			Median of the peak	−0.006 **	0.003	−2.344	−0.511 **
			Mean of the peak	−0.006 **	0.003	−2.106	−0.467 **
		FCR	Median of peak	0.013 *	0.007	1.822	0.392 *
			Mean of peak	0.016 **	0.008	2.039	0.427 **
		FCU	-	-	-	-	-
	Picket	BR	Mean of average	0.001 *	0.001	1.742	0.399 *
			Median of the peak	0.009 *	0.005	1.871	0.380 *
			Mean of the peak	0.011 *	0.006	1.794	0.369 *
		ECU	Median of average	−0.001 *	0.001	−1.947	−0.406 *
			Mean of average	−0.001 **	0.001	−1.985	−0.421 **
			Median of the peak	−0.005 **	0.002	−1.988	−0.437 **
			Mean of the peak	−0.005 **	0.002	−1.957	−0.430 **
		FCR	Median of average	0.001 *	0.001	1.706	0.350 *
			Mean of average	0.001 *	0.001	1.719	0.351 *
			Median of the peak	0.016 **	0.008	1.957	0.417 **
			Mean of the peak	0.016 **	0.008	2.035	0.425 **
		FCU	-	-	-	-	-

*: *p* < 0.1; **: *p* < 0.05; ***: *p* < 0.01; ****: *p* < 0.001.

**Table 4:** Impact of the lower arm dimensions on four muscle activation level measures across 16 groups.

Groups			Lower Arm as a Significant Impact on Muscle Activation Level	Unstandardized Coefficient	Standard Error	*t*-Value	Standardized Coefficient
Expertise	Task	Muscle					
Novice	Deck	BR	Median of average	−0.094 **	0.045	−2.095	−0.437 **
			Mean of average	−0.092 **	0.043	−2.122	−0.448 **
			Median of the peak	−0.369 **	0.178	−2.077	−0.449 **
			Mean of the peak	−0.389 **	0.182	−2.143	−0.457 **
		ECU	Median of average	−0.099 ***	0.032	−3.129	−0.542 ***
			Mean of average	−0.096 ***	0.029	−3.282	−0.556 ***
			Median of the peak	−0.393 ***	0.140	−2.811	−0.579 ***
			Mean of the peak	−0.438 ***	0.155	−2.826	−0.589 ***
		FCR	Median of average	−0.206 **	0.085	−2.42	−0.448 **
			Mean of average	−0.199 **	0.089	−2.228	−0.424 **
			Median of the peak	−0.845 *	0.434	−1.948	−0.384 *
			Mean of the peak	−0.887 *	0.460	−1.930	−0.374 *
		FCU	-	-	-	-	-
	Picket	BR	Median of average	−0.089 ***	0.03	−2.95	−0.581 ***
			Mean of average	−0.091 ***	0.031	−2.956	−0.578 ***
			Median of the peak	−0.530 ****	0.159	−3.335	−0.618 ****
			Mean of the peak	−0.528 ****	0.152	−3.473	−0.643 ****
		ECU	Median of average	−0.136 ****	0.042	−3.220	−0.575 ****
			Mean of average	−0.138 ****	0.042	−3.325	−0.589 ****
			Median of the peak	−0.715 ***	0.249	−2.873	−0.591 ***
			Mean of the peak	−0.755 ***	0.250	−3.016	−0.609 ***
		FCR	Median of average	−0.170 ***	0.062	−2.735	−0.439 ***
			Mean of average	−0.170 ***	0.066	−2.587	−0.427 ***
			Median of the peak	−0.880 **	0.399	−2.207	−0.379 **
			Mean of the peak	−1.066 **	0.481	−2.217	−0.384 **
		FCU	Median of average	−0.066 *	0.035	−1.901	−0.427 *
			Mean of average	−0.066 *	0.035	−1.866	−0.423 *
			Median of the peak	−0.384 **	0.175	−2.192	−0.482 **
			Mean of the peak	−0.383 **	0.173	−2.205	−0.481 **
Expert	Deck	BR	-	-	-	-	-
		ECU	Median of average	−0.047 **	0.023	−2.037	−0.538 **
			Mean of average	−0.044 **	0.023	−2.002	−0.513 **
		FCR	-	-	-	-	-
		FCU	Median of average	−0.048 ***	0.019	−2.588	−0.705 ***
			Mean of average	−0.045 ***	0.017	−2.609	−0.713 ***
			Median of the peak	−0.181 *	0.103	−1.759	−0.516 *
			Mean of the peak	−0.191 **	0.095	−2.012	−0.573 **
	Picket	BR	Mean of average	−0.023 *	0.014	−1.687	−0.478 *
			Median of the peak	−0.670 ***	0.218	−3.072	−0.772 ***
			Mean of the peak	−0.804 ***	0.279	−2.881	−0.733 ***
		ECU	Median of average	−0.048 *	0.025	−1.950	−0.503 *
			Mean of average	−0.047 *	0.025	−1.905	−0.500 *
			Median of the peak	−0.186 *	0.108	−1.723	−0.469 *
			Mean of the peak	−0.188 *	0.107	−1.763	0.479 *
		FCR	-	-	-	-	-
		FCU	Median of average	-0.032 *	0.018	−1.793	−0.507 *
			Mean of average	−0.031 *	0.018	−1.748	−0.501 *

*: *p* < 0.1; **: *p* < 0.05; ***: *p* < 0.01; ****: *p* < 0.001.

**Table 5:** Impact of the upper arm dimensions on the muscle activation level measures across 16 groups.

Groups			The Upper Arm Has a Significant Impact on Muscle Activation Level	Unstandardized Coefficient	Standard Error	*t*-Value	Standardized Coefficient
Expertise	Task	Muscle					
Novice	Deck	BR	-	-	-	-	-
		ECU	-	-	-	-	-
		FCR	-	-	-	-	-
		FCU	-	-	-	-	-
	Picket	BR	-	-	-	-	-
		ECU	Median of the peak	0.230 *	0.139	1.652	0.365 *
			Mean of the peak	0.231 *	0.140	1.649	0.358 *
		FCR	-	-	-	-	-
		FCU	-	-	-	-	-
Expert	Deck	BR	-	-	-	-	-
		ECU	-	-	-	-	-
		FCR	-	-	-	-	-
		FCU	-	-	-	-	-
	Picket	BR	Mean of the peak	0.285 *	0.165	1.724	0.085 *
		ECU	-	-	-	-	-
		FCR	-	-	-	-	-
		FCU	-	-	-	-	-

*: *p* < 0.1; **: *p* < 0.05; ***: *p* < 0.01; ****: *p* < 0.001.
